# Diabetes, Celiac, and Thyroid-Related Autoantibodies in HLA Genotyped Ethiopian Children and Adolescents With Type 1 Diabetes: A Cross-Sectional Study

**DOI:** 10.1155/pedi/8258430

**Published:** 2025-08-17

**Authors:** Adugna Negussie Gudeta, Alexander Lind, Alemayehu Girma, Johanna Lempainen, Jorma Ilonen, Daniel Agardh

**Affiliations:** ^1^Armauer Hansen Research Institute, Addis Ababa, Ethiopia; ^2^Department of Microbiology, Arsi University, Asella, Ethiopia; ^3^Department of Clinical Sciences Malmö, Clinical Research Center, Lund University, Malmo, Sweden; ^4^Department of Pediatrics, Adama Hospital Medical College, Adama, Ethiopia; ^5^Department of Pediatrics, University of Turku, Turku, Finland; ^6^Department of Pediatrics and Adolescent Medicine, Turku University Hospital, Turku, Finland; ^7^Immunogenetics Laboratory, Institute of Biomedicine, University of Turku, Turku, Finland

**Keywords:** autoimmune thyroid disease, celiac disease, human leukocyte antigen, islet autoantibodies, type 1 diabetes

## Abstract

**Background:** Autoantibodies against β-cell components in the pancreatic islets of Langerhans are characteristic of type 1 diabetes (T1D). The genetic and autoimmune determinants of type 1 diabetes (T1D) in Ethiopians are not yet thoroughly characterized, with studies indicating a lower occurrence of autoantibodies related to T1D compared to Caucasians. The study aimed to determine the occurrence of autoantibodies related to type 1 diabetes (T1D), celiac disease (CD), and autoimmune thyroid disease (AITD) in conjunction with Human Leukocyte Antigen (HLA) genotype in Ethiopian children and adolescents with T1D.

**Methods:** This cross-sectional study included 206 children and adolescents with T1D (ranging from 1 to 18 years old) with a median disease duration of 6 years, alongside 200 age-matched control children (ranging from 1 to 6 years old). Participants were recruited from Adama, Asella, and Bishoftu Hospitals in Ethiopia. The study involved genotyping of HLA alleles, specifically HLA-DQA1, DQB1, and DRB1*⁣*^*∗*^04 (including DR4 subtypes). Additionally, autoantibodies targeting glutamic acid decarboxylase (GADA), insulinoma-associated protein (IA-2A), zinc transporter 8 (ZnT8A), tissue transglutaminase (tTGA), and thyroid peroxidase (TPOA) were analyzed using antibody detection by agglutination PCR (ADAP) assays.

**Results:** The most common haplotype found in participants with T1D was HLA-(DR3)-DQA1*⁣*^*∗*^05-DQB1*⁣*^*∗*^02 haplotype (36.4%) (OR = 5.0; *p*  < 0.000001). In addition, HLA-DRB1*⁣*^*∗*^0405-DQA1*⁣*^*∗*^03-DQB1*⁣*^*∗*^02 (19.3%, OR = 10.8; *p*  < 0.000001), HLA-DRB1*⁣*^*∗*^0405-DQA1*⁣*^*∗*^03-DQB1*⁣*^*∗*^0302 (9.2%, OR = 3.1; *p*=0.001), and HLA-DRB1*⁣*^*∗*^0401-DQA1*⁣*^*∗*^03-DQB1*⁣*^*∗*^0302 (3.2%, OR = 20.0; *p*=0.002) were significantly increased among T1D patients. Conversely, HLA-(DR15)-DQB1*⁣*^*∗*^0602, HLA-(DR13)-DQB1*⁣*^*∗*^0603, HLA-(DR1/10)-DQB1*⁣*^*∗*^0501, HLA-(DR13)-DQB1*⁣*^*∗*^0604, HLA-DRB1*⁣*^*∗*^0404-DQA1*⁣*^*∗*^03-DQB1*⁣*^*∗*^04, HLA-(DR7)-DQA1*⁣*^*∗*^0201-DQB1*⁣*^*∗*^02, HLA-(DR11/12/13)-DQA1*⁣*^*∗*^05-DQB1*⁣*^*∗*^0301, and HLA-DRB1*⁣*^*∗*^0403-DQA1*⁣*^*∗*^03-DQB1*⁣*^*∗*^0302 were noted as the most protective haplotypes with a significant *p* value and, with ORs ranging from 0.05 to 0.5. The overall frequency of any islet autoantibodies in children and adolescents with T1D was 81.1% compared to 5.5% in the control group (*p*  < 0.0001). While comparing antibody positivity between individuals with T1D and controls, GADA was found in 69% versus 2%, IA-2A in 24% versus 1.5%, ZnT8A in 32% versus 2%, tTGA in 14% versus 2%, and TPOA in 17% versus 5%, respectively (*p*  < 0.0001). Individuals carrying DR4-DQ8 or DR3-DQ2 haplotypes exhibited a higher prevalence of IA-2A and tTGA (*p* ≤ 0.05).

**Conclusions:** The HLA risk profile typical of sub-Saharan African population was observed in Ethiopians with T1D. Furthermore, they have a notably high prevalence of autoantibodies associated with T1D, CD, and AITD, which differs from earlier reports from the region but aligns with patterns observed in Caucasians.

## 1. Introduction

Type 1 diabetes (T1D) is described by the destruction of the pancreatic β-cell within the islets of Langerhans, resulting in insulin depletion and hyperglycemia [1]. The autoimmune progression to clinical stage 3 T1D can be anticipated by detection of autoantibodies against insulin (IAA), IA-2A, GADA, and ZnT8A. Screening for islet autoantibodies in children with T1D offers crucial insights into the autoimmune mechanisms of the disease. This screening aids in early detection, informs prognosis, guides personalized therapy, and advances our understanding of disease mechanisms, ultimately leading to improved patient outcomes [2]. Although the involvement of autoimmunity in the development of T1D is widely studied worldwide, there is a limited understanding of how this process arises in numerous low- and middle-income countries (LMICs) in sub-Saharan Africa. It remains uncertain whether the underlying mechanisms of the disease in these regions align with those observed in high-income countries, where autoimmune destruction of the pancreatic β-cell is a prominent feature [3].

T1D is closely linked to the HLA-DRB1*⁣*^*∗*^0301-DQA1*⁣*^*∗*^0501-DQB1*⁣*^*∗*^0201 [DR3-DQ2] and HLA-DRB1*⁣*^*∗*^0401/2/4/5-DQA1*⁣*^*∗*^0301-DQB1*⁣*^*∗*^0302 [DR4-DQ8] haplotypes [4]. These haplotypes are found in 58% of children with T1D, whereas only 9% of the general population in Finland, home to the highest T1D incidence worldwide [5]. In Scandinavian countries, the DR4 allele found in the HLA-DRB1*⁣*^*∗*^04-DQA1*⁣*^*∗*^03-DQB1*⁣*^*∗*^0302 [DR4-DQ8] haplotypes is prevalent among both cases and controls. The main variants are DRB1*⁣*^*∗*^0401 and with DRB1*⁣*^*∗*^0404 being less common and less predisposed than DRB1*⁣*^*∗*^0401. In southern Europe and North African countries, the risk-associated HLA-DRB1*⁣*^*∗*^04-DQA1*⁣*^*∗*^03-DQB1*⁣*^*∗*^0302 [DR4-DQ8] haplotypes are primarily represented by DRB1*⁣*^*∗*^0402 and DRB1*⁣*^*∗*^0405. Additionally, the HLA-DRB1*⁣*^*∗*^0405-DQB1*⁣*^*∗*^03-DQB1*⁣*^*∗*^02 [DR4-DQ2] haplotype is also frequently observed [6, 7].

The estimated incidence rates of T1D vary significantly, ranging from 1.5 to 10.1 cases per 100,000 individuals in sub-Saharan Africa, influenced by the country and age group [8]. In 2019, the International Diabetes Federation (IDF) projected that around 25,000 children and adolescents under 20 years old were living with T1D in Africa [9]. However, there is a notable scarcity of comprehensive information from many sub-Saharan African nations, particularly regarding studies on autoantibodies related to T1D. Furthermore, some research indicates a potential link between T1D incidence in African populations and malnutrition [10].

The commonness of celiac disease (CD) and autoimmune thyroid disease (AITD) is greater among individuals with T1D compared to the general population, although the overlap between these three conditions varies by region [11]. This overlap is believed to be due to shared HLA risk genotypes and non-HLA risk loci [12]. Distinct clinical features from the typical presentation of T1D have been observed in African countries, such as reduced occurrence in childhood before puberty, a notable increase in cases during the third decade, significant links to socioeconomic disadvantage, gender disparities with a higher prevalence among males in specific areas, and a reported decrease in the presence of autoantibodies [13, 14].

The study sought to fill the existing knowledge gap by determining the occurrence of islet-, celiac-, and thyroid-associated autoantibodies in HLA-genotyped Ethiopian participants with T1D.

## 2. Materials and Methods

### 2.1. Study Setting

The research was carried out at three hospitals in southeastern Ethiopia: Adama Medical College Hospital (about 100 km from Addis Ababa), Bishoftu Hospital (about 60 km from Addis Ababa), and Asella Referral and Teaching Hospital (around 175 km from Addis Ababa). These facilities provide comprehensive healthcare services to both rural and urban communities within all age groups.

### 2.2. Study Design and Population

The cross-sectional study encompassed 206 patients attending pediatric diabetic clinics across three hospitals. The study participants were predominantly male (63.6%), with an average age of 10.89 ± 4.62 years. Participants with T1D were enrolled consecutively during routine follow-up visits, ensuring an unbiased sample collection. Inclusion criteria included all T1D patients under 18 years old attending the pediatric and diabetic clinics. All participants had been on continuous insulin therapy since diagnosis, suggesting that any observed IAAs were likely induced by insulin therapy rather than the disease process.

 Structured interviews with parents were carried out using standardized questionnaires to gather detailed clinical histories, family histories of autoimmune disorders, and demographic information. Trained nurses conducted physical examinations and anthropometric measurements to assess participants' health status and growth metrics. Furthermore, blood samples were collected from each participant to screen for T1D, CD, and AITD-associated autoantibodies.

The study incorporated a control group of 200 children, of whom 54% were male, selected randomly from the existing “Traditional Ethiopian Food (TEF)” birth cohort in Adama. These controls originated from a population-based study focused on identifying environmental and immunogenetic factors associated with CD in Ethiopia. Residual samples from the TEF cohort, stored in a repository, were randomly chosen and analyzed using laboratory methods comparable to those used for the T1D group, ensuring consistency for accurate comparison [15].

### 2.3. Sample Collection

Venous blood samples were collected from all participants during their routine clinical follow-up for T1D. The serum was separated on the same day as collection and stored at −80°C, following the same protocol for the control group before analysis. All samples from both groups were processed under standardized conditions to ensure consistency and maintain the reliability of the results.

### 2.4. Multiplex Antibody Detection by Agglutination PCR (ADAP) Technology

Autoantibodies targeting GAD, IA-2, ZnT8, tTG, and TPO were quantified using ADAP as previously described at the Clinical Research Center (CRC), Lund University, Sweden [16, 17]. Autoantibody levels were expressed as ΔCt, and cut-offs were determined from readouts in quantile quantile plots.

### 2.5. HLA Typing

HLA genotyping was performed stepwise, starting with DQB1 genotyping. If the identified DQB1 alleles were associated with multiple different DQA1-DQB1 haplotypes, additional typing was conducted to analyze DQA1 alleles. Genotyping also included specific HLA-DRB1*⁣*^*∗*^04 alleles that are associated with DQA1*⁣*^*∗*^03-DQB1*⁣*^*∗*^0301, DQA1*⁣*^*∗*^03-DQB1*⁣*^*∗*^0302, and DQA1*⁣*^*∗*^03-DQB1*⁣*^*∗*^02 haplotypes. DRB1-DQA1-DQB1 haplotypes listed in Tables 1 and 2 include DRB1 alleles in parentheses when they are consistently found with the genotyped alleles. The genotyping procedure has been described in detail in reference [5].

### 2.6. Statistical Analysis

All data analyses were conducted using IBM SPSS Statistics software, version 28.0 (IBM Corp., Armonk, NY, USA), Prism version 10.2.3 (GraphPad Software, San Diego, CA, USA), and MedCalc (MedCalc Software Ltd., Ostend, Belgium). Categorical variables were compared using chi-square tests or Fisher's exact tests. Multivariable logistic regression analyses were employed to assess differences in multiple autoantibodies across different age categories and genders. Categorical data were summarized as percentages and frequencies. The normality of autoantibody distributions was assessed using quantile–quantile (Q–Q) normality plots. For comparisons of mean autoantibody levels, independent samples *t*-tests were performed. A *p* value below 0.05 was regarded as statistically significant.

### 2.7. Ethics

The Institutional Review Board of Adama Hospital Medical College approved this study. Informed consent was obtained from all parents or guardians, and assent was obtained from adolescent participants. All samples and associated questionnaires were coded to maintain confidentiality, with no identifying personal information recorded.

## 3. Results

Children and adolescents with T1D carried most often HLA-(DR3)-DQA1*⁣*^*∗*^05-DQB1*⁣*^*∗*^02, with 13.9% being homozygous for this haplotype. HLA-DRB1*⁣*^*∗*^0405-DQA1*⁣*^*∗*^03-DQB1*⁣*^*∗*^02, DRB1*⁣*^*∗*^0405-DQA1*⁣*^*∗*^03-DQB1*⁣*^*∗*^0302, and DRB1*⁣*^*∗*^0401-DQA1*⁣*^*∗*^03-DQB1*⁣*^*∗*^0302 were also significantly more frequent among cases with T1D compared to controls (Table 1). In cases with T1D, the most common HLA-DR4 haplotype was associated with the DRB1*⁣*^*∗*^0405 allele, followed by DRB1*⁣*^*∗*^0401. In contrast, haplotypes, such as HLA-DR15-DQB1*⁣*^*∗*^0602, HLA-DR13-DQB1*⁣*^*∗*^0603, HLA-(DR1/10)-DQB1*⁣*^*∗*^0501, HLA-(DR13)-DQB1*⁣*^*∗*^0604, HLA-DRB1*⁣*^*∗*^0404-DQA1*⁣*^*∗*^03-DQB1*⁣*^*∗*^04, HLA-DRB1*⁣*^*∗*^0403-DQA1*⁣*^*∗*^03-DQB1*⁣*^*∗*^02, HLA-(DR7)-DQA1*⁣*^*∗*^0201-DQB1*⁣*^*∗*^02, HLA-(DR7)-DQA1*⁣*^*∗*^0201-DQB1*⁣*^*∗*^0303, HLA-DR11/12/13-DQA1*⁣*^*∗*^05-DQB1*⁣*^*∗*^0301, and HLA-DRB1*⁣*^*∗*^0403-DQA1*⁣*^*∗*^03-DQB1*⁣*^*∗*^0302 were less frequent with odds ratios ranging from 0.02 to 0.13 (Table 1).

Genotypes with significant disease association included homozygosity for HLA-(DR3)-DQA1*⁣*^*∗*^05-DQB1*⁣*^*∗*^02 as well as its combinations with DRB1*⁣*^*∗*^0405-DQA1*⁣*^*∗*^03-DQB1*⁣*^*∗*^0302, DRB1*⁣*^*∗*^0405-DQB1*⁣*^*∗*^03-DQB1*⁣*^*∗*^02, DRB1*⁣*^*∗*^0401-DQB1*⁣*^*∗*^0302, and (DR7)-DQA1*⁣*^*∗*^02: 01-DQB1*⁣*^*∗*^02 haplotypes. Also, a combination of DRB1*⁣*^*∗*^0405-DQB1*⁣*^*∗*^03-DQB1*⁣*^*∗*^02 and DRB1*⁣*^*∗*^0405-DQA1*⁣*^*∗*^03-DQB1*⁣*^*∗*^0302 and combinations of DRB1*⁣*^*∗*^0405-DQA1*⁣*^*∗*^03-DQB1*⁣*^*∗*^02 with (DR1/10)-DQB1*⁣*^*∗*^0501 and DR13 DQB1*⁣*^*∗*^0604 haplotypes were also more frequent in the T1D group compared to controls (Table 2). The HLA-DRB1*⁣*^*∗*^0401-DQA1*⁣*^*∗*^03-DQB1*⁣*^*∗*^0302 haplotype, associated with the greatest risk in Northern Europe, is rare in sub-Saharan Africa. It was not detected in any of the control groups; however, 11 haplotypes were identified within the T1D group.

**Figure 1:**
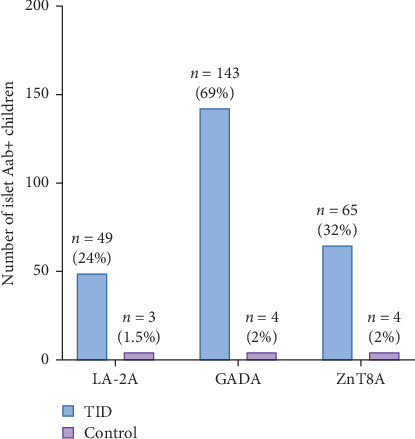
Prevalence of islet autoantibodies in type 1 diabetes (T1D) patients and controls.

**Figure 2:**
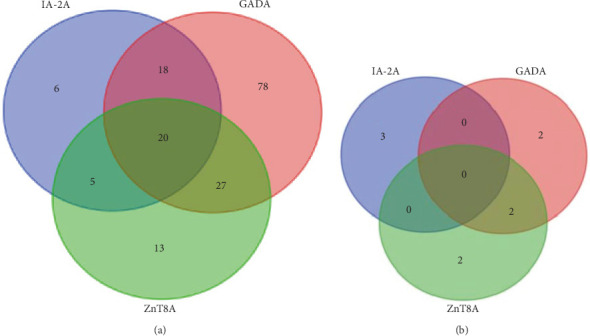
Venn diagrams of islet autoantibody profiles in type 1 diabetes (T1D) patients (A) and controls (B).

The occurrence of islet autoantibodies in individuals with T1D and controls is presented in Figure 1 and Supporting Information Figure S1. Among T1D subjects, 69% were positive for GADA, 32% were positive for ZnT8A, and 24% were positive for IA-2A, compared to 2% positivity in the control group for each autoantibody (GADA, ZnT8A, IA-2A) (*p* < 0.05). The overall prevalence of any islet autoantibody in subjects with T1D, excluding IAA, was 81%, with 34% testing positive for multiple autoantibodies. A significant coexistence between ZnT8A and GADA was observed in T1D patients (*p*=0.033, Figure 2).

The positivity of each autoantibody was analyzed in relation to HLA-DR-DQ genotypes. A significant association with GADA was observed in the following genotypes: HLA-(DR3)-DQA1*⁣*^*∗*^05-DQB1*⁣*^*∗*^02/(DR3)-DQA1*⁣*^*∗*^05-DQB1*⁣*^*∗*^02, HLA-(DR3)-DQA1*⁣*^*∗*^05-DQB1*⁣*^*∗*^02/DRB1*⁣*^*∗*^0405-DQA1*⁣*^*∗*^03-DQB1*⁣*^*∗*^0302, HLA-(DR3)-DQA1*⁣*^*∗*^05-DQB1*⁣*^*∗*^02/DRB1*⁣*^*∗*^0405-DQB1*⁣*^*∗*^03-DQB1*⁣*^*∗*^02, HLA-(DR3)-DQA1*⁣*^*∗*^05-DQB1*⁣*^*∗*^02/X, and DRB1*⁣*^*∗*^0405-DQA1*⁣*^*∗*^03-DQB1*⁣*^*∗*^0302/X. In addition, IA-2A and ZnT8A were significantly related to HLA-(DR3)-DQA1*⁣*^*∗*^05-DQB1*⁣*^*∗*^02/X and DRB1*⁣*^*∗*^0405-DQA1*⁣*^*∗*^03-DQB1*⁣*^*∗*^0302/X, respectively (Table 2).

**Figure 3:**
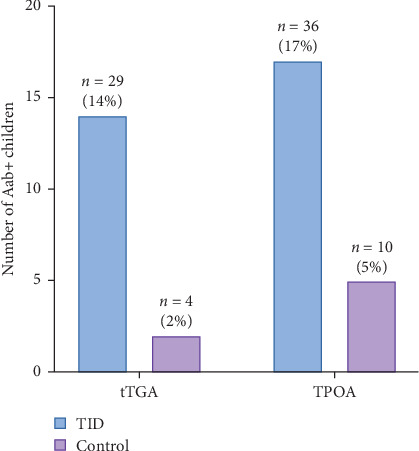
Prevalence of celiac disease autoimmunity (i.e., tissue transglutaminase autoantibodies, tTGAs) and thyroid autoimmunity (i.e., thyroperoxidase autoantibodies, TPOAs) in type 1 diabetes (T1D) patients and controls.

The data highlights a significantly higher prevalence of tTGA and TPOA among children and adolescents with T1D compared to controls. Specifically, 14% of T1D patients tested positive for tTGA, and 17% for TPOA, whereas only 2% and 5%, respectively, of controls showed positivity (*p*=0.0001) (Figure 3). Nearly all individuals with positive tTGA results possess at least one of the designated high-risk haplotypes: HLA-(DR3)-DQA1*⁣*^*∗*^05-DQB1*⁣*^*∗*^02, DRB1*⁣*^*∗*^0405-DQB1*⁣*^*∗*^03-DQB1*⁣*^*∗*^02, or DRB1*⁣*^*∗*^0405-DQA1*⁣*^*∗*^03-DQB1*⁣*^*∗*^0302. Notably, among the TPOA-positive T1D participants, 91% also possessed one of these haplotypes (Table 3).

The coexistence of all five screened autoantibodies (GADA, IA-2A, ZnT8A, tTGA, and TPOA) measured by ADAP was observed in 2 (1%) individuals with T1D compared to none in controls (not significant). A statistically significant coexistence of tTGA and TPOA (*p*=0.001) and GADA and TPOA (*p* < 0.001) was noted among T1D participants (Supporting Information Figure S2).

The study revealed an age-related variation in IA-2A (*p*=0.02) and tTGA (*p*=0.03) levels (Supporting Information Figure S3). Additionally, among individuals testing positive for tTGA, higher tTGA level were linked to male (*p*=0.034) (Supporting Information Figure S3). No correlations were found between autoantibody positivity and BMI (Supporting Information Table S1).

## 4. Discussion

This comprehensive study conducted in Ethiopia used newly developed ADAP assays to assess the frequency of autoantibodies associated with T1D, CD, and AITD. The findings revealed that the majority of individuals exhibited these autoantibodies alongside previously described risk alleles linked to T1D. Conversely, a smaller subset of participants tested negative for autoantibodies and showed some minor differences in their immunological profiles. Notably, the age range of the T1D participants was between 1 and 18 years, which is below the typically reported highest incidence age range of 20−29 years in the wider African population [18]. The study also highlights the limited research on HLA class II genetic markers associated with autoimmunity in the Ethiopian context. Most prior studies have examined only a limited number of such markers. However, both this current research and the earlier TEF study birth cohort [15], comprising a relatively large number of T1D cases, have included detailed analyses of HLA DR4 subtypes and haplotypes, focusing on the DRB1, DQA1, and DQB loci. These genetic investigations provide valuable insights into the immunogenetic factors influencing T1D risk within this population.

Consistent with previous global studies on T1D cases, the current research showed the predominance of HLA-(DR3)-DQA1*⁣*^*∗*^05-DQB1*⁣*^*∗*^02 haplotype, which is the most prevalent and highest risk-linked haplotype, followed by DRB1*⁣*^*∗*^0405-DQA1*⁣*^*∗*^03-DQB1*⁣*^*∗*^02 and DRB1*⁣*^*∗*^0405-DQA1*⁣*^*∗*^03-DQB1*⁣*^*∗*^0302. The (DR3)-DQA1*⁣*^*∗*^05-DQB1*⁣*^*∗*^02 haplotype is common across most populations with T1D, from Northern Europe to sub-Saharan Africa, while DRB1*⁣*^*∗*^0405-DQA1*⁣*^*∗*^03-DQB1*⁣*^*∗*^02 is typical in sub-Saharan Africa, Egypt, and Saudi Arabia, though rare in Northern Europe, where DRB1*⁣*^*∗*^0401-DQA1*⁣*^*∗*^03-DQB1*⁣*^*∗*^0302 is the major T1D-associated haplotype, alongside DRB1*⁣*^*∗*^0404-DQA1*⁣*^*∗*^03-DQB1*⁣*^*∗*^0302 [5, 6, 19]. These haplotypes remain relatively rare in Africa and Southern Europe, but the association with risk is still clear. Additionally, DRB1*⁣*^*∗*^0402-DQA1*⁣*^*∗*^03-DQB1*⁣*^*∗*^0302 was observed only in one Ethiopian sample, whereas it is relatively common in Southern Europe and Egypt. Conversely, DRB1*⁣*^*∗*^0405-DQA1*⁣*^*∗*^03-DQB1*⁣*^*∗*^0302 was found to be relatively common in T1D cases and strongly associated with significant risk. This haplotype is rare in Northern Europe but more common in Southern Europe, Saudi Arabia, and Egypt, where it confers a strong risk for disease development [6, 7, 19]. Beyond the risk-associated haplotypes, the research also identified several known protective haplotypes, such as (DR15)-DQB1*⁣*^*∗*^0602, (DR11/12/13)-DQA1*⁣*^*∗*^05-DQB1*⁣*^*∗*^0301, (DR13)-DQB1*⁣*^*∗*^0603, (DR7)-DQA1*⁣*^*∗*^0201-DQB1*⁣*^*∗*^0303, and DRB1*⁣*^*∗*^0403-DQA1*⁣*^*∗*^03-DQB1*⁣*^*∗*^0302. Haplotypes with lesser protective effects, including (DR1/10)-(DQA1*⁣*^*∗*^)-DQB1*⁣*^*∗*^0501, (DR7)-DQA1*⁣*^*∗*^0201-DQB1*⁣*^*∗*^02, and (DR13)-DQB1*⁣*^*∗*^0604, were the most frequent in the control group, a pattern that closely resembles findings from an Egyptian population [6].

Concerning genotype-level risk, we found the highest risk associated with (DR3)-DQA1*⁣*^*∗*^05-DQB1*⁣*^*∗*^02/DRB1*⁣*^*∗*^0405-DQA1*⁣*^*∗*^03-DQB1*⁣*^*∗*^02 and (DR3)-DQA1*⁣*^*∗*^05-DQB1*⁣*^*∗*^02/DRB1*⁣*^*∗*^0405-DQA1*⁣*^*∗*^03-DQB1*⁣*^*∗*^0302 heterozygotes, which combine two risk haplotypes. The (DR3)-DQA1*⁣*^*∗*^05-DQB1*⁣*^*∗*^02/(DR7)-DQA1*⁣*^*∗*^0201-DQB1*⁣*^*∗*^02 combination had a high OR value, but because of the small number of cases, this resulted in a high confidence interval. (DR3)-DQA1*⁣*^*∗*^05-DQB1*⁣*^*∗*^02 homozygotes show lower OR values, supporting the highest risk of heterozygous genotypes with two different risk haplotypes, as observed in several other populations [6]. DR3 homozygotes were the most common genotype observed among children with T1D in Ethiopia, as reported globally. Conversely, homozygosity for DRB1*⁣*^*∗*^0405-DQA1*⁣*^*∗*^03-DQB1*⁣*^*∗*^0302 is rare among T1D patients, and only a few heterozygous individuals with DRB1*⁣*^*∗*^0405-DQA1*⁣*^*∗*^03-DQB1*⁣*^*∗*^0302/DRB1*⁣*^*∗*^0405-DQA1*⁣*^*∗*^03-DQB1*⁣*^*∗*^02 were observed in patients, with none found in controls, highlighting their potential role in increasing disease risk. Most children with T1D possessed a combination of two risk haplotypes. This finding is consistent with a similar study conducted on Egyptian children with T1D [6]. Additionally, this study indicated that genotypes carrying protective haplotypes were not frequent among Ethiopian children with T1D.

The current study reports a notably higher overall prevalence of any islet autoantibodies (81%) among individuals with T1D in the sub-Saharan African population compared to previously reported figures [14, 20, 21]. This prevalence aligns closely with data from European and Mediterranean populations, where autoantibody prevalence typically ranges from 80% to 97% [22]. It is important to highlight that IAA were omitted from this analysis because all tests were conducted after patients had begun insulin therapy, making it difficult to distinguish between autoantibodies related to the disease and those generated by insulin treatment.

The study highlights that GADA is the most frequently detected autoantibody in individuals, with T1D compared to control groups, consistent with prior research involving Ethiopian children and adolescents [23]. GADA remains among the most common autoantibodies associated with T1D across diverse populations, including those in Africa, Europe, and America [3, 14, 24, 25]. A notable finding is that over 25% of children with T1D in the current cohort who possess specific HLA genotypes, particularly homozygosity for alleles like (DR3)-DQA1*⁣*^*∗*^05-DQB1*⁣*^*∗*^02 or certain heterozygous combinations, such as (DR3)-DQA1*⁣*^*∗*^05-DQB1*⁣*^*∗*^02/DRB1*⁣*^*∗*^0405-DQA1*⁣*^*∗*^03-DQB1*⁣*^*∗*^0302 or (DR3)-DQA1*⁣*^*∗*^05-DQB1*⁣*^*∗*^02/DRB1*⁣*^*∗*^0405-DQA1*⁣*^*∗*^03-DQB1*⁣*^*∗*^02, tested positive for GADA. Furthermore, children carrying genotypes, such as HLA-(DR3)-DQA1*⁣*^*∗*^05-DQB1*⁣*^*∗*^02/X or DRB1*⁣*^*∗*^0405-DQA1*⁣*^*∗*^03-DQB1*⁣*^*∗*^0302/X tested positive for GADA. These findings suggest a strong relation between specific HLA genotypes and the presence of GADA in pediatric T1D patients, indicating a genetic predisposition linked to autoimmune mechanisms.

ZnT8A is a marker that may be particularly useful in assigning risk from adolescence onwards [26]. However, data on the frequency of ZnT8A and IA-2A in the African population are scarce. The current study found a higher prevalence of ZnT8A compared to the control and previous research conducted in the African region, including a study previously conducted in Ethiopia [23, 24, 27]. The rate of ZnT8A is lower compared to the prevalence reported in the Caucasian population, where it was observed in two-thirds of individuals with T1D at the time of diagnosis [14, 23, 27]. A significant association with ZnT8A autoantibody positivity was seen in DRB1*⁣*^*∗*^0405-DQA1*⁣*^*∗*^03-DQB1*⁣*^*∗*^0302/X genotypes.

IA-2A was identified in about one-fourth of the participants with T1D, significantly more often than in the control group. The research demonstrated a notable correlation with individuals carrying the HLA-(DR3)-DQA1*⁣*^*∗*^05-DQB1*⁣*^*∗*^02/X genotype. This finding is contrast with prior research from the same region, which noted either an absence or a low frequency of IA-2A in the Ethiopian T1D population [23, 25]. The current prevalence of IA-2A is also higher compared to reports from other sub-Saharan African countries [14]. However, it is still lower than the prevalence of IA-2A reported in industrialized nations [27].

The study findings indicate that 34% of islet autoantibody-positive individuals also tested positive for multiple autoantibodies, with a significant association observed specifically between GADA and ZnT8A. While variations exist among different autoantibody combinations, these results align with most previous studies. Notably, some studies have identified a significant link between ZnT8A and IA-2A [28], suggesting their involvement in the later stages of disease progression. These findings challenge the common perception that islet autoantibodies are either absent or rare in individuals with T1D within sub-Saharan African populations. Additionally, this study found no differences in islet-related autoantibody prevalence based on gender.

The observed differences between this study and earlier sub-Saharan African studies can be attributed to a complex interplay of various factors. These include variations in the average duration of diabetes at the time of testing, differences in participants' age at screening, and disparities in study population size. Additionally, genetic factors such as HLA genotype distributions, environmental influences, and differences in assay techniques can all impact results. The timing of the study, whether conducted at diagnosis or after prolonged insulin therapy, along with the diagnostic methods employed and the overall study design, also play crucial roles. Furthermore, the underlying causes of T1D may differ between industrialized countries and sub-Saharan Africa, potentially influencing autoantibody prevalence rates.

The hallmarks of AITD, TPOA, were analyzed in the current study and were detected in one out of six participants with T1D over an average monitoring duration of 6 ± 5 years. It was also observed that TPOAs are more common in female participants. This result is consistent with findings from Syria (16%) [29], Iraq (17%) [30], Kuwait (15%) [31], Egypt (12%) [32], and Italy (12%) [33]. However, the prevalence observed in the present research was lower than that documented in India (24%) [34], Iran (40%) [35], Korea (26%) [36], Armenia (25%) [37], and Brazil (25%) [38]. Additionally, the current study's findings exceed those of studies conducted in Iran (9%), [39], Sudan (6%) [40], Cameroon (4%), [21], and Sweden (7%) [41]. The study also found that most TPOA-positive participants carried DQB1*⁣*^*∗*^02 or DQB1*⁣*^*∗*^0302.

In the current study, the prevalence of tTGA was 14% among the participants with T1D. This was seven times higher than in the controls selected from the previous birth cohort [15]. This result aligns with findings from other worldwide studies of CD-associated autoantibodies among T1D patients, such as those from India (14%) [42], Saudi Arabia (10%) [43], Algeria (16%–20%) [44], and Iraq (11%) [45]. In contrast, the current finding is higher than the results from Turkey (4%) [46] and Jordan (6%) [47]. A significant proportion of tTGA positivity was observed in young children less than 10 years old. Of the 28 T1D individuals who tested positive for tTGA, 24 (83%) had at least one additional islet autoantibody or TPOA. All but one of these individuals tested positive for (DR3)-DQA1*⁣*^*∗*^05-DQB1*⁣*^*∗*^02, except for the person who carried both DRB1*⁣*^*∗*^0405-DQA1*⁣*^*∗*^03-DQB1*⁣*^*∗*^02 and  DRB1*⁣*^*∗*^0405-DQA1*⁣*^*∗*^03-DQB1*⁣*^*∗*^0302 haplotypes. This highlights the significance of DR3-DQ2-associated autoantibodies in the pathogenesis of CD [48].

The observed disparities in the frequency of TPOA and tTGA across different studies can be attributed to multiple factors. These include variations in HLA distribution among populations, differences in study designs and methodologies, demographic and population heterogeneity, sample sizes, testing protocols, and criteria used to interpret seropositivity. Additionally, differences in underlying pathogenic etiologies, diagnostic assays employed, duration and stage of illness, as well as environmental influences, contribute to the variability.

This study possesses several notable strengths. Primarily, the serological data were analyzed using ADAP, one of the most advanced and sensitive techniques currently available, enhancing the reliability of the findings. It is also the first study to simultaneously assay five autoantibodies within a sub-Saharan African population, providing a comprehensive immunological profile. Furthermore, the interpretation of results was based on locally established healthy controls rather than relying solely on manufacturer-provided cut-off values, thereby increasing the specificity and relevance of the findings.

In addition, this research is pioneering in examining the co-occurrence of three common autoimmune disorders within the region, offering valuable insights into regional disease patterns. This study offers strong evidence indicating the contribution of HLA in the pathogenesis of T1D, based on a detailed analysis of HLA-DR-DQ alleles in both the study patients and controls.

The study included certain limitations. Firstly, autoantibodies such as islet autoantibodies, tTGA, and TPOA were only screened once, which limits understanding of their persistence over time and their status at the time of diagnosis. Longitudinal data would be necessary to clarify their role in disease progression. Furthermore, the study's limited geographic scope may constrain the applicability of these findings to the wider Ethiopian population. An additional significant limitation is the variation in age distributions between the cases and controls.

## 5. Conclusion

This study concludes that Ethiopian patients with T1D display HLA-DR-DQ haplotype risk patterns characteristic of sub-Saharan African populations, alongside a high prevalence of islet autoantibodies, typically linked to findings that contrast with earlier regional reports but align more closely with data from Caucasian populations. These findings reaffirm the autoimmune nature of T1D in Ethiopia. Moreover, the markedly higher occurrence of TPOA and tTGA in Ethiopian T1D patients compared to controls underlines the prominence of implementing routine screening for AITD and CD in children and adolescents with T1D. Overall, these findings provide a useful baseline for the prompt detection of individuals at risk of developing T1D and associated autoimmune disorders within this population.

## Figures and Tables

**Table 1:** Frequency of HLA-DR-DQB haplotypes in T1D patients and controls.

HLA-DRB1*⁣*^*∗*^-DQA1*⁣*^*∗*^-DQB1*⁣*^*∗*^ haplotype	T1D group *N* = 202 (100%)	Control group *N* = 166 (100%)	OR	95% CI	*p*-Value
0401-03-0302	11 (3.2)	0	20.0	2.6–155.4	0.002
0405-03-02	78 (19.3)	7 (2.1)	10.8	5.1–22.6	<0.000001
(03)-05-02	147 (36.4)	33 (9.9)	5.0	3.3–7.5	<0.000001
0404-03-0302	5 (1.2)	1 (0.3)	4.1	0.8–20.7	NS
0405-03-0302	37 (9.2)	10 (3.0)	3.1	1.6–6.3	0.001
0410-03-02	1 (0.2)	0	2.4	0.2–26.6	NS
(x)-0502/3	1 (0.2)	0	2.4	0.2–26.6	NS
0402-03-0302	1 (0.2)	0	2.4	0.2–26.6	NS
(16)-0502	1 (0.2)	0	2.4	0.2–26.6	NS
(x)-03-02	5 (1.2)	2 (0.6)	2.0	0.5–8.1	NS
(08)-(01)-04	7 (1.7)	6 (1.8)	0.9	0.33–2.6	NS
0401-03-0301	2 (0.5)	2 (0.6)	0.8	0.16–4.1	NS
(09)-03-0303	0	1 (0.3)	0.3	0.01–6.7	NS
(14)-0503	0	1 (0.3)	0.3	0.01–6.7	NS
0403-03-02	0	6 (1.8)	0.06	0.0–1.1	NS
0404-03-04	0	7 (2.1)	0.05	0.0–0.9	0.04
(15)-(01)-0602	0	23 (6.9)	0.02	0.0–0.3	0.05
(01/10)-(01)-0501	38 (9.4)	51 (15.4)	0.5	0.3–0.8	0.0008
(13)-(01)-0609	9 (2.2)	15 (4.5)	0.5	0.2–1.0	0.07
(13)-(01)-0604	26 (6.4)	39 (11.7)	0.5	0.3–0.8	0.008
(07)-0201-02	26 (6.4)	51 (15.4)	0.4	0.2–0.6	0.00003
(07)-0201-0303	2 (0.5)	7 (2.1)	0.2	0.06–0.9	0.04
0403-03-0302	2 (0.5)	12 (3.6)	0.13	0.04–0.4	0.002
(13)-(01)-0603	2 (0.5)	17 (5.1)	0.09	0.03–0.3	0.00006
(11/12/13)-05-0301	3 (0.7)	41 (12.3)	0.05	0.02–0.1	<0.000001

Abbreviations: OR, odds ratio; T1D, type 1 diabetes.

**Table 2:** Frequency of HLA-DR-DQB genotypes in T1D patients and controls.

HLA-DRB1*⁣*^*∗*^-DQA1*⁣*^*∗*^-DQB1*⁣*^*∗*^/DRB1*⁣*^*∗*^-DQA1*⁣*^*∗*^-DQB1*⁣*^*∗*^ genotype	T1D group *N* = 202 (100%)	Control group *N* = 166 (100%)	OR	95% CI	*p*-Value
(03)-05-02/0405-03-02	25 (12.4)	1 (0.6)	23.3	5.45–99.7	0.00001
(03)-05-02/(07)-0201-02	11 (5.5)	0	20.0	2.6–155.4	0.0004
(03)-05-02/0405-03-0302	20 (9.9)	1 (0.6)	18.1	4.2–78.5	0.0001
0405-03-02/(13)-0604	6 (3.0)	0	11.0	1.3–90.4	0.025
(01/10)-0501/0405-03-02	11 (5.5)	1 (0.6)	6.6	1.5–30.0	0.009
(03)-05-02/(03)-05-02	28 (13.9)	5 (3.0)	5.2	2.1–12.8	0.0003
0405-03-02/0405-03-0302	5 (2.5)	0	9.3	1.1–77.8	0.04
(03)-05-02/0401-03-0302	5 (2.5)	0	9.3	1.1–77.8	0.04
(13)-0604/(03)-05-02	3 (1.5)	0	5.8	0.7–52.8	NS
(08)-04/0405-03-02	3 (1.5)	0	5.9	0.7–53.6	NS
0405-03-02/(13)-0609	3 (1.5)	0	5.9	0.7–53.6	NS
0405-03-02/0404-03-0302	3 (1.5)	0	5.9	0.7–53.6	NS
0405-03-0302/(01/10)-0501	5 (2.5)	1 (0.6)	4.2	0.8–21.0	NS
0405-03-02/0405-03-02	5 (2.5)	2 (1.2)	2.1	0.5–8.4	NS
(03)-05-02/(01/10)-0501	9 (4.5)	4 (2.4)	1.9	0.6–5.6	NS
(03)-05-02/(13)-0604	9 (3.0)	4 (2.4)	1.9	0.6–5.6	NS
(13)-0604/(13)-0604	3 (1.5)	2 (1.2)	1.2	0.3–5.6	NS
(07)-0201-02/0405-03-0302	3 (1.5)	2 (1.2)	1.2	0.3–5.6	NS
(01/10)-0501/(01/10)-0501	3 (1.5)	5 (3.0)	0.5	0.1–1.7	NS
(01/10)-0501/(07)-0201-02	3 (1.5)	7 (4.2)	0.3	0.1–1.2	NS
Others	39 (19.3)	131 (78.9)	—	—	—

Abbreviations: NS, not significant; OR, odds ratio; T1D, type 1 diabetes.

**Table 3:** Frequencies of HLA-DRB-DQB1 genotypes in T1D patients by autoantibody type.

HLA-DRB1*⁣*^*∗*^-DQA1*⁣*^*∗*^-DQB1*⁣*^*∗*^/DRB1*⁣*^*∗*^-DQA1*⁣*^*∗*^-DQB1*⁣*^*∗*^ genotype	Total	IA-2A+ *n* (%)	GADA+ *n* (%)	ZnT8A+ *n* (%)	tTGA+ *n* (%)	TPOA+ *n* (%)	No islet antibodies *n* (%)
(03)-05-02/0405-03-02	25	3 (12.0)	15 (60.0)*⁣*^*∗*^	7 (28.0)	3 (12.0)	4 (16.0)	7 (28.0)
(03)-05-02/(07)-0201-02	11	4 (36.4)	6 (54.5)	4 (36.4)	1 (9.1)	2 (18.2)	4 (36.4)
(03)-05-02/(03)-05-02	28	6 (21.4)	21 (75.0)*⁣*^*∗*^	8 (28.6)	6 (21.4)	5 (17.9)	2 (7.1)
(03)-05-02/0405-03-0302	20	6 (30.0)	14 (70.0)*⁣*^*∗*^	6 (30.0)	1 (5.0)	2 (10.0)	5 (25.0)
0405-03-02/X	24	7 (29.2)	13 (54.2)	8 (33.3)	2 (8.3)	2 (8.3)	1 (4.2)
0405-03-02/0405-03-0302	5	2 (40.0)	2 (40.0)	2 (40.0)	1 (20.0)	0 (0.0)	0 (0.0)
(03)-05-02/0401-03-0302	5	0 (0.0)	2 (40.0)	0 (0.0)	0 (0.0)	2 (40.0)	1 (20.0)
(03)-05-02/X	26	7 (26.9)	17 (65.4)*⁣*^*∗*^	10 (38.5)*⁣*^*∗*^	3 (11.5)	6 (23.1)	5 (19.2)
0405-03-02/0405-03-02	6	1 (16.7)	4 (66.7)	2 (33.3)	0 (0.0)	1 (16.0)	0 (0.0)
0405-03-02/0401-03-0302	4	0 (20.4)	2 (50.0)	0 (0.0)	0 (0.0)	2 (50.0)	1 (25.0)
0405-03-0302/X	15	7 (46.7)*⁣*^*∗*^	13 (86.7)*⁣*^*∗*^	6 (40.0)	5 (33.3)*⁣*^*∗*^	3 (20.0)	0 (0.0)
(07)-0201-02/0405-03-0302	4	0 (0.0)	3 (75.0)	0 (0.0)	0 (0.0)	2 (50.0)	0 (0.0)
(07)-0201-02/0405-03-02	3	2 (66.7)	3 (100.0)	1 (33.3)	0 (0.0)	1 (33.3)	0 (0.0)
(03)-05-02/0404-03-0302	2	0 (0.0)	1 (50.0)	0 (0.0)	0 (0.0)	0 (0.0)	0 (0.0)
(03)-05-02/0403-03-0302	2	1 (50.0)	2 (100.0)	1 (50.0)	0 (0.0)	0 (0.0)	0 (0.0)
(07)-0201-02/X	8	2 (25.0)	3 (37.5)	1 (12.5)	0 (0.0)	0 (0.0)	1 (12.5)
X/X	14	3 (21.4)	10 (71.4)	6 (42.9)	3 (21.4)	3 (21.4)	2 (12.3)
Total	202	48	139	63	27	34	39

*Note:* X, any haplotypes other than DRB1*⁣*^*∗*^0405-DQA1*⁣*^*∗*^03-DQB1*⁣*^*∗*^0302, (DR3)-DQA1*⁣*^*∗*^05-DQB1*⁣*^*∗*^02, HLA-DRB1*⁣*^*∗*^0405-DQA1*⁣*^*∗*^03-DQB1*⁣*^*∗*^02, DRB1*⁣*^*∗*^0401-DQA1*⁣*^*∗*^03-DQB1*⁣*^*∗*^0302 (DR4-DQ8), and HLA-(DR7)-DQA1*⁣*^*∗*^0201-DQB1*⁣*^*∗*^02.

*⁣* ^*∗*^ *p* ≤ 0.05.

## Data Availability

The data underlying the findings of this study are provided within the article. For further information or questions about the data, please contact the corresponding author.

## Supporting Information

Supporting Information 1 Figure S1: Compare the pattern of multiple islet autoantibodies in individuals with T1D and the control group.

Supporting Information 2 Figure S2: Illustrates comparison of TPOA, tTGA, and three islet autoantibodies measured by ADAP in T1D patients and control groups.

Supporting Information 3 Figure S3: Average distribution of autoantibodies across different age groups in children with T1DM.

Supporting Information 4 Figure S4: Compares the mean antibody levels for males and females for each antibody.

Supporting Information 5 Table S1: The table provides a comparative overview of various characteristics between children with T1D and a control group.
