# Supplementary figures and images for: Diabetes, Celiac, and Thyroid-Related Autoantibodies in HLA Genotyped Ethiopian Children and Adolescents With Type 1 Diabetes: A Cross-Sectional Study
Source: Pediatr Diabetes. 2025 Aug 17;2025:8258430. doi: 10.1155/pedi/8258430 (PMC12375835; doi:10.1155/pedi/8258430)

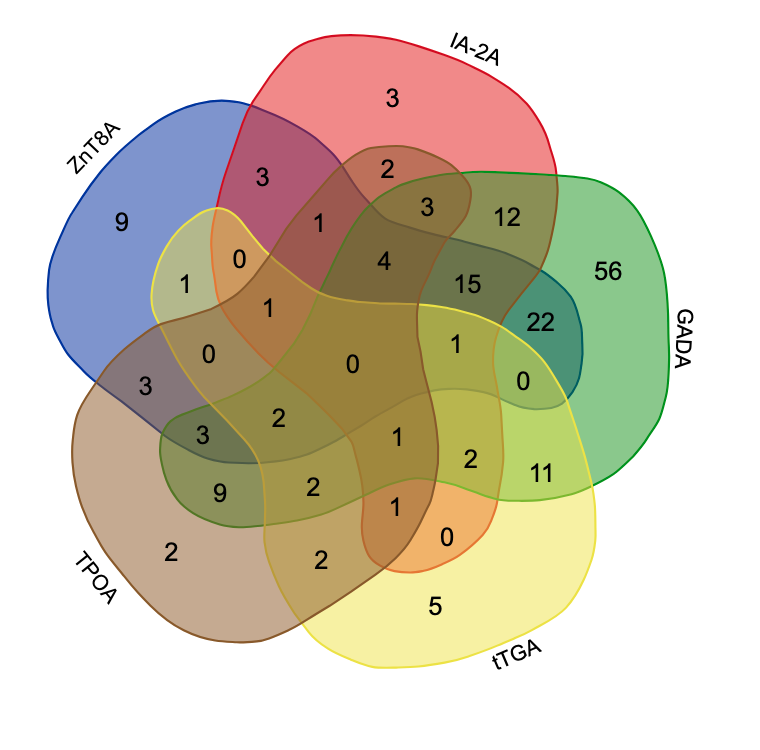
**
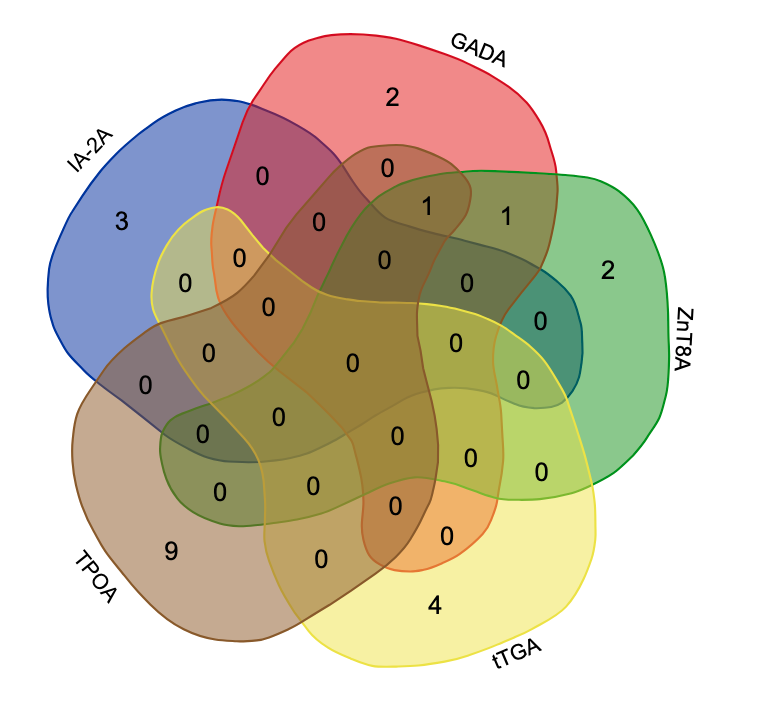
**

(A) (B)

Supplement: Supporting Information 2 — Figure S2: Illustrates comparison of TPOA, tTGA, and three islet autoantibodies measured by ADAP in T1D patients and control groups. [file 8258430.f2.docx]

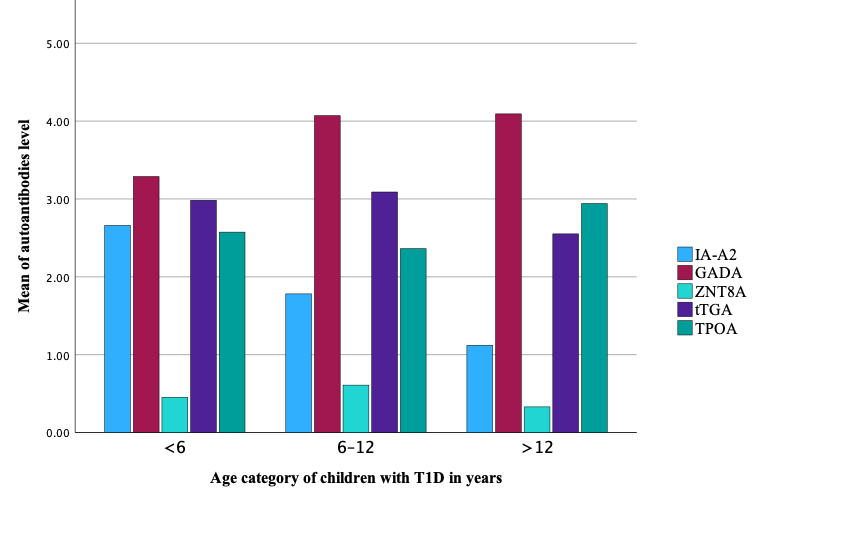

Supplement: Supporting Information 3 — Figure S3: Average distribution of autoantibodies across different age groups in children with T1DM. [file 8258430.f3.docx]

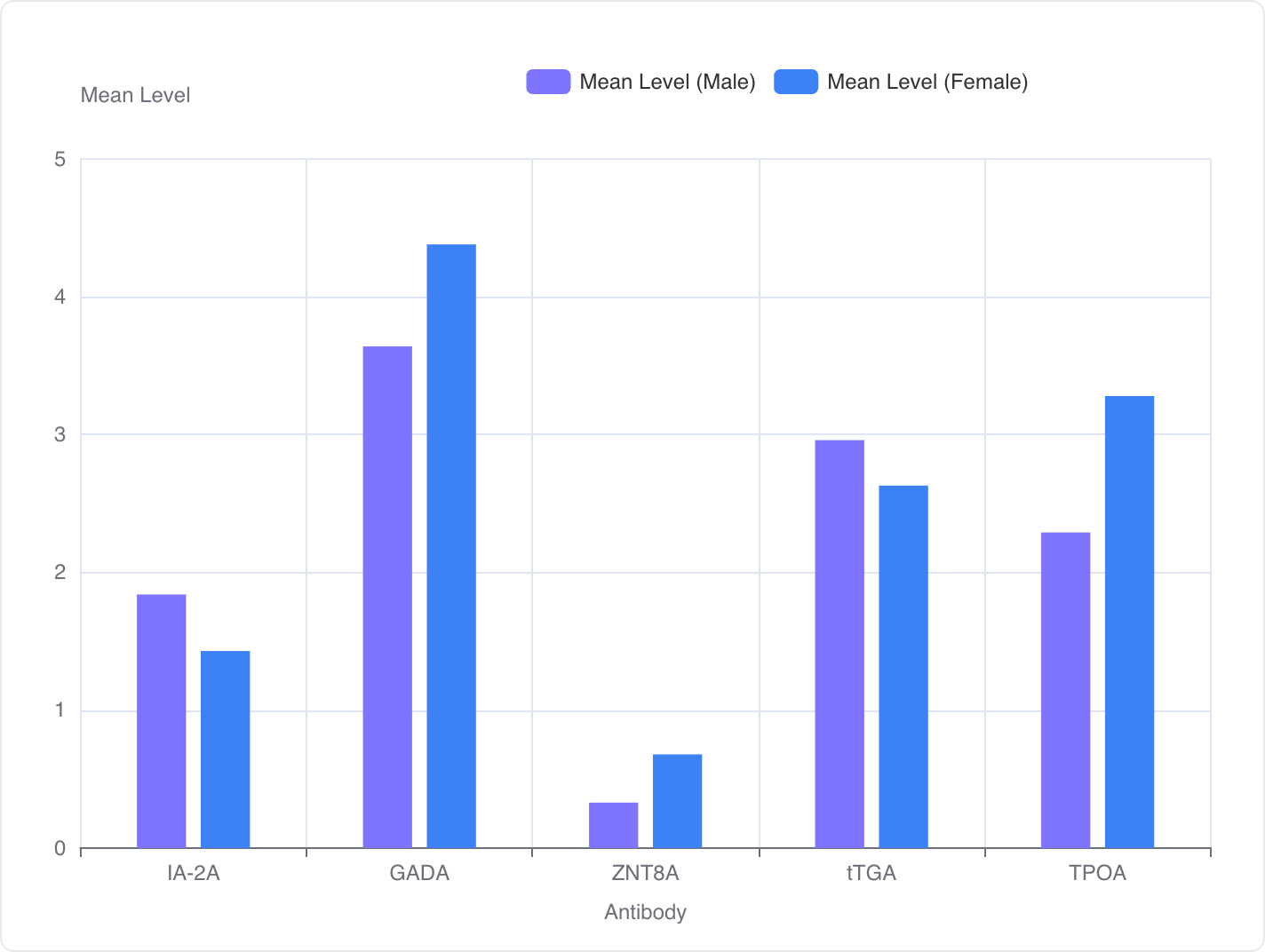

Supplement: Supporting Information 4 — Figure S4: Compares the mean antibody levels for males and females for each antibody. [file 8258430.f4.docx]
