# Supplementary material for: Diabetes, Celiac, and Thyroid-Related Autoantibodies in HLA Genotyped Ethiopian Children and Adolescents With Type 1 Diabetes: A Cross-Sectional Study
Source: Pediatr Diabetes. 2025 Aug 17;2025:8258430. doi: 10.1155/pedi/8258430 (PMC12375835; doi:10.1155/pedi/8258430)
Supplement: Supporting Information 5 — Table S1: The table provides a comparative overview of various characteristics between children with T1D and a control group. [file 8258430.f5.docx]

**Supplementary Table 1. Demographic characteristics of the study subjects**

The table provides a comparative overview of various characteristics between children with T1D and a control group. In terms of sex distribution, the T1D group comprises 63.6% males, whereas the control group includes 54% males. The mean age of children with T1D is about 10.89 years (SD ± 4.62), with age categories showing that 21.4% are aged 6 years or younger, 36.9% are between 6 and 12 years, and 41.7% are over 12 years. In contrast, the control group’s mean age is 4 years (SD ± 4.4), although specific age categories are not detailed for this group. Regarding delivery methods, 12.6% of children with T1D were born via Caesarean section, while the remaining 87.4% had spontaneous deliveries. The average BMI among T1D children is 16.74 (SD ± 4.01), which is higher than the control group's mean BMI of 14.67 (SD ± 1.55). The duration of diabetes in the T1D group averages approximately 6.15 years (SD ± 4.91), and the mean age at disease onset is about 4.8 years (SD ± 3.7). Notably, 22.8% of T1D children have a family history of diabetes, whereas data regarding family history is not provided for controls. Additionally, most T1D participants (68.9%) reside in urban areas, with the remaining 31.1% living in rural settings.

| Characteristics | T1D (n=206) | Control (n=200) |
| --- | --- | --- |
| Sex (Male/Female), n (%) | 131 (63.6)/75 (36.4) | 108 (54)/92 (46) |
| Age in years (mean ± SD)  Age category: ≤6 years old  6–12  >12 | 10.89 ± 4.62  44 (21.4%)  76 (36.9%)  86 (41.7%) | 4 ± 4.4  200  ––  –– |
| Child Delivery methods: Caesarean section  Spontaneous | 26 (12.6%)  180 (87.4%) |  |
| Body mass index, mean ±SD | 16.74 ± 4.01 | 14.67 ± 1.55 |
| Duration of diabetes, years (mean ± SD) | 6.15 ± 4.91 | –––––––– |
| Average age of T1D onset (mean ± SD) | 4.8 ± 3.7 | ––––––– |
| Family History for DM, % Yes/No | 22.8/77.2 | ––––––––– |
| Residence of study participants, % City/rural | 68.9/31.1 | –––––––––– |
