# Supplementary material for: Diabetes, Celiac, and Thyroid-Related Autoantibodies in HLA Genotyped Ethiopian Children and Adolescents With Type 1 Diabetes: A Cross-Sectional Study
Source: Pediatr Diabetes. 2025 Aug 17;2025:8258430. doi: 10.1155/pedi/8258430 (PMC12375835; doi:10.1155/pedi/8258430)
Supplement: Supplementary file 3 [file 8258430.f1.docx]

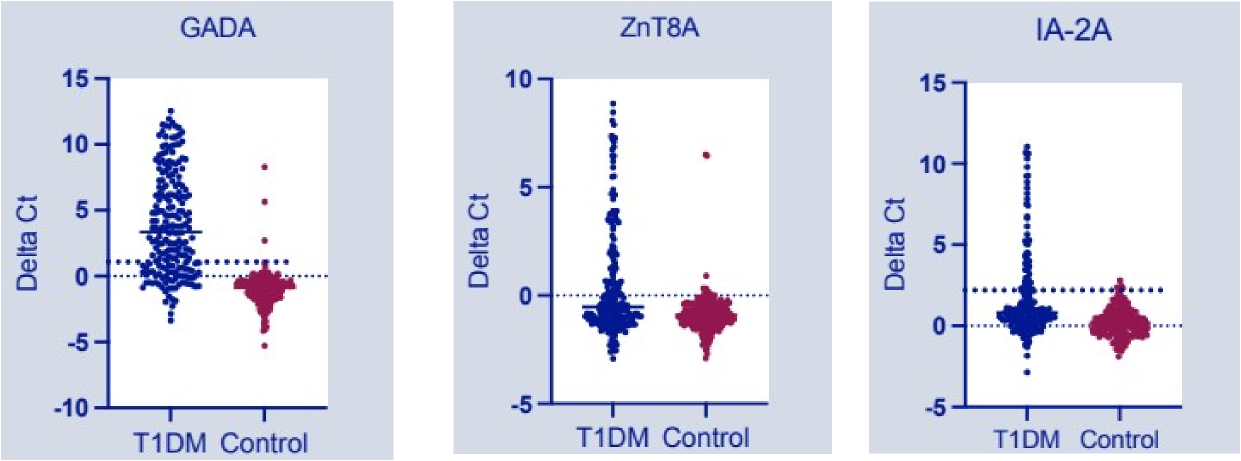
GADA
Delta Ct
15
10
5
0
-5
-10
T1DM
Control
ZnT8A
Delta Ct
10
5
0
-5
T1DM
Control
IA-2A
Delta Ct
15
10
5
0
-5
T1DM
Control
